# Measurement Matrix Construction for Large-area Single Photon Compressive Imaging

**DOI:** 10.3390/s19030474

**Published:** 2019-01-24

**Authors:** Hui Wang, Qiurong Yan, Bing Li, Chenglong Yuan, Yuhao Wang

**Affiliations:** 1 School of Information Engineering, Nanchang University, Nanchang 330031, China; wanghuiyy35@163.com (H.W.); iamlibing_417816@163.com (B.L.); 13361646796@163.com (C.Y.); wangyuhao@ncu.edu.cn (Y.W.); 2 State Key Laboratory of Transient Optics and Photonics, Xi’an Institute of Optics and Precision Mechanics, Chinese Academy of Sciences, Xi’an 710119, China

**Keywords:** single photon compressive imaging, compressed sensing, measurement matrix

## Abstract

We have developed a single photon compressive imaging system based on single photon counting technology and compressed sensing theory, using a photomultiplier tube (PMT) photon counting head as the bucket detector. This system can realize ultra-weak light imaging with the imaging area up to the entire digital micromirror device (DMD) working region. The measurement matrix in this system is required to be binary due to the two working states of the micromirror corresponding to two controlled elements. And it has a great impact on the performance of the imaging system, because it involves modulation of the optical signal and image reconstruction. Three kinds of binary matrix including sparse binary random matrix, m sequence matrix and true random number matrix are constructed. The properties of these matrices are analyzed theoretically with the uncertainty principle. The parameters of measurement matrix including sparsity ratio, compressive sampling ratio and reconstruction time are verified in the experimental system. The experimental results show that, the increase of sparsity ratio and compressive sampling ratio can improve the reconstruction quality. However, when the increase is up to a certain value, the reconstruction quality tends to be saturated. Compared to the other two types of measurement matrices, the m sequence matrix has better performance in image reconstruction.

## 1. Introduction

Photon counting imaging is a method of imaging at very low light levels based on photon counting technology, which thus has broad application prospects in the field of ultra-weak light detection, such as biomedical detection [1,2,3,4], deep space exploration [5], and spectral measurement [6,7]. At present, the detectors with single photon counting capability like photoelectric multiplier, avalanche photodiode and superconducting single-photon detector, all belong to single-pixel detector. Therefore, to obtain the spatial resolution, the optical scanning equipment is required to scan the imaging space point by point [8,9]. Unfortunately, this approach has disadvantages in scanning time, efficiency of photon collection and resolution of time [10]. Though detector arrays like intensified CCD (ICCD) and electron-multiplying CCD (EMCCD), can work in photon counting mode, the cost of CCD development is high because of the requirement of an extremely high readout frame frequency and the low circuit noise [5,11,12,13]. However, more and more types of single photon-counting detector arrays are developed to obtain spatial information in recent years, such as SPAD arrays, microchannel plate photomultipliers (MCP-PMTs) [14,15], multi anode photomultipliers (MAPMTs) [16], silicon photomultiplier (SIPM) arrays [17,18] and so on. Besides, more recently complementary metal-oxide-semiconductor (CMOS) single-photon avalanche diode (SPAD) arrays have emerged as candidates for solid-state image sensors for single-photon imaging applications [19,20,21]. 

With the introduction of compressed sensing theory, a new imaging method called single-pixel imaging appeared in 2008 [22,23,24]. In this scheme, the image is randomly modulated by a DMD, and the two-dimensional image is reconstructed with the optical signal received by a single-pixel detector each time and the pseudo-random code loaded into DMD. Compared to the above imaging by the point-by-point scanning or the detector array, the point detector receives the total light intensity of multiple pixels, so that the larger luminous flux can be collected by the single-pixel detector. Therefore, the imaging sensitivity of the single-pixel imaging system is no longer limited by the detection sensitivity of single photon detector, and the larger signal-to-noise ratio (SNR) is obtained. In addition, single-pixel imaging is well suited for situations in which sensitive array detectors are either technically or financially unavailable at the target wavelength bands [25].

Since the typical detector working in analog mode, converts the detected optical signal into current signal and the precision is poor [24], there is no obvious advantage for single-pixel imaging using in strong light detection. Super sensitivity of photon counting imaging can be realized by utilizing single photon detector, due to the good linear relationship of output photon counts and incident light intensity at ultra-weak light levels [9,26,27]. In 2012, Yu of the Chinese Academy of Sciences combined the single-pixel imaging technology with single photon counting technology, first realize super sensitive imaging in a very small area. Obviously higher sensitivity than photon counting imaging based on the single photon detector array is verified experimentally [28]. This method, which uses the advantages of high flux and sub-sampling of compressive imaging and high sensitivity of photon counting to achieve ultra-sensitive imaging, is called single photon compressive imaging. In the same year Howell et al. proposed a compressive depth image acquisition scheme based on the single photon-counting detector [29,30]. In 2016, Dai et al. demonstrated a photon counting 3D imaging system with a single-pixel photon counting detector [31,32]. However, the imaging schemes in those papers only used a small region of DMD, and the imaging area is very small. This has led to difficulties in large-aperture, wide-field imaging.

Single photon compressive imaging has the ability to perform compressive sampling and image reconstruction. Measurement matrix, sparse representation of signal, signal reconstruction are three core links of compressive sensing. Due to participating in two important processes of compressive sensing (CS), compressive sampling and signal recovery, the measurement matrix has a great influence on the performance of the whole imaging system. In 2015, Tiwari et al. designed the sparse sensing matrix to obtain high resolution medical images with least incurred computational cost [33]. In 2017, Obermeier et al. described a method for designing sensing matrix to improve CS recovery capabilities in electromagnetic imaging applications [34]. In this paper, a large-area single photon compressive imaging system is constructed. Compared to systems in the existing literature, which only use a small region of DMD, this system can realize imaging with the image plane up to the entire DMD work region. Three different binarization measurement matrices are constructed. Then the performance of the single photon compressive imaging system under different measurement matrices is evaluated.

## 2. Principle and Realization of Experimental System

**Figure 1:**
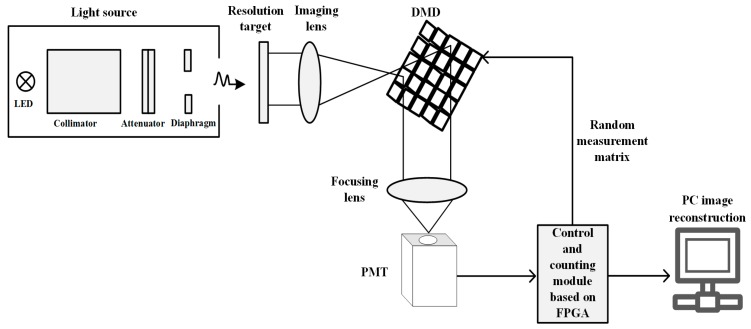
Schematic diagram of single photon compressive imaging system, including a light source, a resolution target, an imaging lens, the digital micromirror device (DMD), a focusing lens, the photomultiplier tubes (PMT), the specially designed circuit for the control of the DMD and counting photons synchronously, and a computer for image reconstruction.

The schematic diagram of single photon compressive imaging system is demonstrated in Figure 1. A parallel light source is specially designed to reduce the influence of light scattering on imaging resolution. The light source is composed of led lamp, collimator, attenuator and diaphragm, which outputs ultra-weak parallel light. The resolution target is illuminated by the extremely weak parallel light and then imaged on the DMD by an imaging lens. The digital microscopy device (0.7XGA 12° DDR DMD, TI, Dallas, TX, USA) consists of a 1024 × 768 micro-mirrors. The size of a micro-mirror is 13.68 μm × 13.68 μm, and each mirror can be controlled to rotate through +12 degrees or −12 degrees independently. A binary random matrix loaded into DMD can control the direction of each micro-mirror simultaneously and realize the modulation of the input light. In the experiment, the modulated light is collected into a single photon detector through a lens set along the +12 degrees’ reflecting direction of micro-mirror. In order to effectively collect the target image covering the whole DMD working region, the detector adopts the photon counting mode photomultiplier with large area (H10682, Hamamatsu Photonics, Hamamatsu, Japan), which has an effective photosensitive area of Φ8 mm. We developed the control and counting module based on FPGA. It can not only load *M* random binary patterns into DMD sequentially to achieve random modulation of the image on DMD, but also can synchronously record the number of photons collected by the detector. Then these photon numbers corresponding to the intensity of modulated light are sent to the computer for imaging reconstruction. The image is reconstructed via compressive sensing reconstruction algorithm.

In the experiment, turn a row vector *Φ_i_* of the measurement matrix Φ into a two-dimensional pattern each time, then load it into DMD for image modulation. So, if representing the image on the DMD with a column vector *x* of *N* dimensions, the measured value *y_i_*, the number of photons received by the detector, can be expressed as the scalar product of signal *x* with row vector *Φ_i_*:

(1) $$y_{i}=\Phi_{i}x$$

The above process in which the DMD loads a frame measurement matrix *Φ_i_* and synchronously records the number of photons detected by the single-pixel detector is called a measurement. A photon count value *y_i_*, i.e., the measured value, is obtained in one measurement. After M measurements, *M*-dimensions measured value *y* is obtained. Then considering the system noise, the entire measurement process can be expressed as Equation (2):

(2) y=Φx+e

where Φ has the dimensions of *M* × *N* and e is the measurement noise. The image *x* can be recovered by using the *M*-dimensions measured value *y* and the measurement matrix Φ. If *M* < *N*, there are infinite solutions for Equation (2) since the number of linear equations is smaller than that of unknowns. However, if the original signal *x* is sparse or sparsified in a certain basis set Ψ, i.e., *x* = Ψ*α*, where *α* is a sparse signal with very few nonzero elements, it’s very likely for the signal *x* to be accurately recovered with a small number of measurements. The problem becomes solving the following equation:

(3) y=ΦΨα+e

*A^CS^* of size *M* × *N*, defined as *A^CS^* = ΦΨ, is called a sensing matrix or information operator of compressive sensing. The reconstruction of signal x can be accomplished by solving the *L0* norm:

(4) $$\min_{x}{\left\|\alpha \right\|}_{0}\;s.t.\;A^{CS}\alpha =y$$

The solution of Equation (4) can be obtained by CS algorithm. A common CS algorithm is the greedy algorithm based on *L0* norm, including orthogonal matching pursuit (OMP), regularized orthogonal matching pursuit (ROMP) and so on. This algorithm chooses the most matching atoms as the support set in the complete atomic library and makes a series of local optimal individual updates to solve the *L0* norm problem [5]. Thus, lower complexity of the algorithm and faster reconstruction speed.

## 3. Construction of Measurement Matrix and Theoretical Analysis

According to the principle of imaging system, measurement matrix plays a key role in image compressive sampling and signal reconstruction. Since the DMD in system requires a binary matrix control the direction of micro-mirror, three kinds of binary measurement matrices are constructed, sparse binary random matrix, m sequence matrix and true random number matrix. With the comparison of classical Gaussian random matrix, the performance of three matrices is analyzed in theory.

### 3.1. Construction of Measurement Matrix

As mentioned above, the imaging area of our system is the entire DMD working region, which corresponding to the micromirrors array of 1024 × 768. However, the compressive imaging of 1024 × 768 requires a very long sampling and reconstruction time. Therefore, a mask construction scheme is proposed to shorten imaging time. In this scheme, the masks loaded into DMD with size of 1024 × 768 is composed of *M* × *N* matrix units, and the elements of each matrix unit are all “0” or “1”. So the mirrors arrays of DMD corresponding to one matrix unit are combined together as one pixel. By this, the imaging of the entire DMD working region is transformed into the imaging of fewer pixels *M* × *N* and a fast large-area imaging is achieved. This scheme has been described in detail in Ref. [35]. Different kinds of measurement matrix corresponding to fewer-pixels imaging is constructed as follow.

To construct the Gaussian random matrix, generate a matrix Φ of size *M* × *N*. Each element is made to independently obey the Gaussian distribution with mean zero and variance 1/*M*. Owing to being incoherent to most orthogonal basis, it can perform accurate reconstruction from a small number of measurements and thus is widely used [36].

To construct the sparse binary random matrix, first generate a zero matrix Φ of size *M* × *N*, then make *d* elements selected randomly be “1” for each column of Φ. Because of the simple structure, sparse binary random matrix is easy to construct and save in practice [37].

The m sequence matrix is a deterministic measurement matrix generated based on the maximum length linear feedback shift register sequence (m sequence). The m sequence is widely used due to good autocorrelation and cross-correlation. And m sequence matrix is composed of two preferred pairs added one bit after cycle shift, the size of which is *M* × *N* (*M* = *N*/2) [37,38].

The true random number matrix in this paper consists of the true random number sequence generated by a self-developed optical quantum random number generator. It extracts the random bits from continuously measuring arrival time of photons with a common starting point. The unbiased and post-processing free random bits obtained have passed all tests in the statistical test suite [39,40].

### 3.2. Theoretical Analysis of Matrix Performance

For accurately reconstructing original signal with high probability in the process of compressive sensing, the sensing matrix *A^CS^* needs to satisfy the restricted isometry property (RIP) according to CS theory [41,42,43]. In other words, the measurement matrix is required to be as incoherent as possible to the sparse basis. Due to the complexity of RIP theory verification, scholars have proposed many other theories to measure the ability of measurement matrix to recover sparse signals [44,45]. Among these theories, the uncertainty principle can measure the performance of measurement matrix in a relatively simple manner. It takes the mutual coherence of the sensing matrix *A^CS^* as the measurement index, which is obtained by computing the maximum cross-correlations coefficient between any two normalized column vectors of the matrix [46]. 

**Figure 2:**
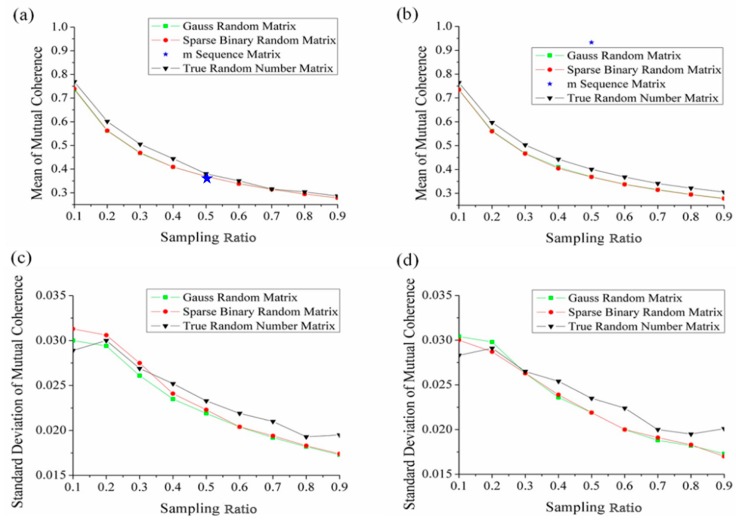
The test results of mutual coherence of the sensing matrices composed by Gauss random matrix, sparse binary random matrix, m sequence matrix and true random number matrix. 10,000 times’ independent experiments are performed on each test point. (**a**) and (**b**) are the mean values of mutual coherence versus sampling ratio when the sparse basis is a discrete cosine basis and a wavelet base respectively. (**c**) and (**d**) are the standard deviation of mutual coherence versus sampling ratio when the sparse basis is a discrete cosine basis and a wavelet base respectively.

In the experiment, the mutual coherence of the sensing matrices composed of the above four measurement matrices is tested respectively when the discrete wavelet basis and the discrete cosine basis are selected as the sparse basis respectively. In order to make the comparison more convincing, we perform 10,000 independent experiments on each test point. The mean and the standard deviation of the mutual coherences versus compressive sampling ratio are shown in Figure 2. Here the compressive sampling ratio is defined as the ratio of the number of measurements to the number of pixels.

Since the size of matrix can’t be changed, the results of the m sequence matrix are given only when the compressive sampling ratio is 0.5. As shown in Figure 2a,b, for the other three types of measurement matrices, the mean of mutual coherence decreases with the increase of sampling ratio. Especially in Figure 2b, when the sampling ratio is 0.5, mutual coherence corresponding to m sequence matrix is 0.9329, which is significantly greater than that of the other three measurement matrices. The mutual coherence of the sensing matrix composed of Gauss random matrix and sparse binary random matrix at different sampling ratio is basically the same (the dotted lines in the figure overlap), and it is less than that of true random number matrix. Figure 2c,d shows the standard deviation of mutual coherence corresponding to true random number matrix is larger when the sampling ratio is greater than 0.3.

## 4. Experimental Performance Verification

In the process of compressive sensing, the performance of measurement matrix is closely related to the matrix sparsity ratio (the proportion of non-zero elements) and the compressive sampling ratio. The performance of the measurement matrix can be evaluated via reconstruction quality and reconstruction time. For a quantitative comparison of the image quality, we introduce three commonly used full-reference evaluation indexes, the mean square error (MSE), peak signal to noise ratio (PSNR), and mean structure similitary index (MSSIM). In order to evaluate the resolution of reconstructed image, the United States Air Force (USAF) test target is used for imaging experiments. Image reconstruction is achieved by OMP algorithm. In experiments, the DMD micro-mirror array is divided into 256 × 256 pixels and each pixel unit has a micro-mirror array of size 4 × 3. So the sampling and reconstruction time of large-area imaging is significant reduced. In the experiment, the photon counting rate of the detector is of the order of 10^3^, which can be used as a characterization of experimental light intensity. 

### 4.1. Influence of Sparsity Ratio on Imaging Quality

**Figure 3:**
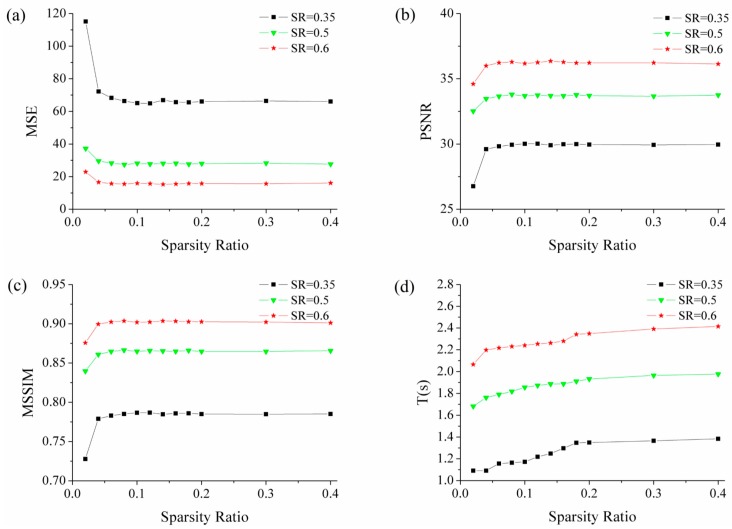
The effect of matrix sparsity ratio on its performance. With sampling ratio of 0.35, 0.5, and 0.6, the parameters MSE (**a**), PSNR (**b**), MSSIM (**c**) and reconstruction time T (**d**) change with sparsity ratio of measurement matrix. Each group of experiments is repeated 500 times.

Taking the sparse binary random matrix for example to analyze the influence of the sparsity ratio on its performance, Figure 3 presents the reconstruction results of the different sparsity ratio measurement matrix. The sampling ratios are taken 0.35, 0.5, 0.6 and each group of experiments is repeated 500 times.

As shown in Figure 3, reconstruction quality is improved with the increase of sparsity ratio when the sparsity ratio of matrix is small. But for a larger sparsity ratio (greater than 0.1), the reconstruction quality stays almost unchanged. The image reconstruction time increases with higher sparsity ratio. Therefore, there is a tradeoff between reconstruction quality and reconstruction time when the sparsity ratio of measurement matrix is small. With the increase of the sparsity ratio (greater than 0.1), the sparsity ratio of the matrix should be reduced as much as possible to shorten the reconstruction time. But in the practical application of single photon compressive imaging system, it is necessary to ensure that the fluctuation of luminous flux per measurement is greater than that of noise fluctuation when reducing the sparsity ratio of measurement matrix. It should be noted that, the reconstruction time shown in Figure 3, which is obtained by the OMP algorithm, is only a reference for the influence of measurement matrix sparsity ratio on reconstruction time. There are many fast and effective reconstruction algorithms for applications that require high real-time performance.

### 4.2. Influence of Compressive Sampling Ratio on Imaging Quality

**Figure 4:**
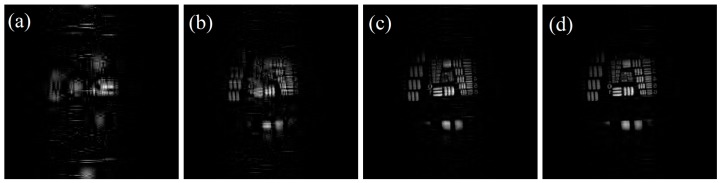
Reconstruction results under different compressive sampling ratios. (**a**–**d**) are reconstruction results with the sampling ratio of 0.1, 0.25, 0.5 and 0.6. The image size is 256 × 256 pixels. The measurement matrix is sparse binary random matrix.

**Figure 5:**
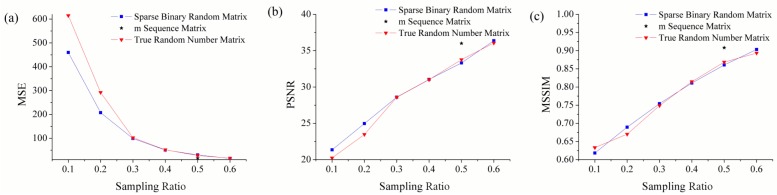
The corresponding curves of evaluation indices with the changing compressive sampling ratio. (**a**) The MSE of reconstructed image with the changing compressive sampling ratio. (**b**) The PSNR of reconstructed image with the changing compressive sampling ratio. (**c**) The MSSIM of reconstructed image with the changing compressive sampling ratio.

The reconstruction results are given in Figure 4 with the compressive sampling ratio of 0.1, 0.25, 0.5 and 0.6 when measurement matrix is sparse binary random matrix. The results show the advantage of compressive sensing in recovering images with a few measurements. The larger the compressive sampling ratio, the better the quality of the reconstructed image. When the sampling ratio is greater than 0.6, the image quality is not significantly improved as the sampling ratio increases. As is mentioned previously, evaluation indices help to quality the reconstructed image under different measurement matrixes. The corresponding curves of evaluation indices with the changing compressive sampling ratio are provided in Figure 5. When the compressive sampling ratio is less than 0.3, the performance of true random number matrix is poor. Whereas the performance of three measurement matrices has little difference when the compressive sampling ratio is greater than 0.3. With the increase of compressive sampling ratio, the performance of true random number matrix is improved, which is consistent with theoretical analysis in Section 3.2.

### 4.3. Comparison of Imaging Performance of Different Measurement Matrices

**Figure 6:**
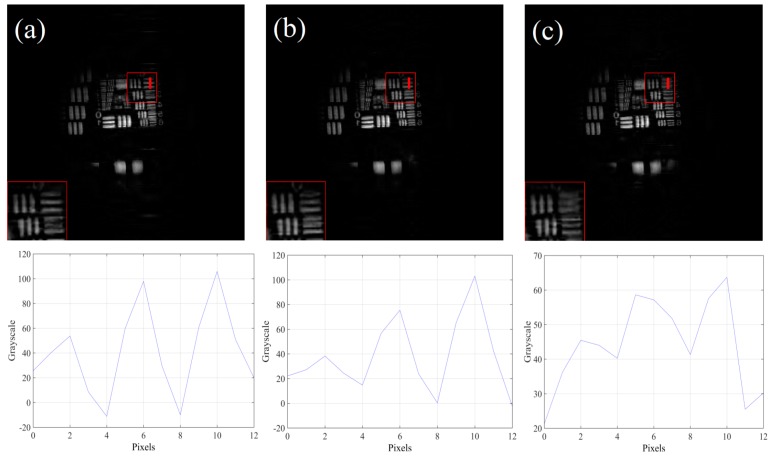
The reconstructed results under different measurement matrices, and the vertical grayscale change curves are given correspondingly. (**a**) Sparse binary random matrix. (**b**) m sequence matrix. (**c**) True random number matrix. The compressive sampling ratios are 0.5.

The evaluation indices of the reconstructed image under different measurement matrices are given in Table 1. The reconstruction results of sparse binary random matrix and true random number matrix are taken as the average value of 500 times, and the compressive sampling ratio is 0.5. From Table 1, it can be seen that m sequence matrix has the best performance, true random number matrix is second. In order to intuitively compare the effect of matrix reconstruction, Fig. 6 shows the reconstructed results under different measurement matrices. To observe edges of the reconstructed image, the vertical grayscale change curves are given correspondingly (Element 2, Group 0 of the resolution target).

Comparing reconstructed images in Figure 6, the resultant reconstruction of the sparse binary random matrix and the m sequence matrix is better than that of the true random number matrix. Combined with the corresponding grayscale curves, it indicates that the pair of lines on the reconstructed images under the sparse binary random matrix and the m sequence matrix can be resolved. However, under true random number matrix, the corresponding pair of lines can’t be resolved and the discontinuity of grayscale change appears.

### 4.4. Influence of Poisson Noise on Imaging Quality

Since the following is an analysis from the perspective of the detector, the Poisson noise related image quality is assumed to be independent of measurement matrix. As the illumination on the object is at the single photon level, the number of photons detected by the single photon detector over a period of time *t* obeys the Poisson distribution. That is to say, the probability of detecting *n* photons in time interval t is:

(5) $$p\left(n,t\right)=\frac{{\left(\lambda t\right)}^{n}e^{-\lambda t}}{n!}=\frac{w^{n}e^{-w}}{n!}$$

where w=λt is the average photons detected in interval *t*. In the imaging experiment, the pulse count output *N_t_* by the detector is the sum of the signal photon number *N_p_*, and the dark count *N_d_*. The detected signal photon count *N_p_* can be expressed as:

(6) $$N_{p}=N_{t}-N_{d}$$

Poisson shot noise *n* in our imaging system is:

(7) $$n=\sqrt{N_{t}}=\sqrt{N_{p}+N_{d}}$$

The signal-to-noise ratio (SNR) of imaging systems is:

(8) $$SNR=\frac{N_{p}}{\sqrt{N_{p}+N_{d}}}$$

If dark count rate is *R_d_*, the SNR will be

(9) $$SNR=\frac{\lambda t}{\sqrt{\lambda t+R_{d}t}}=\frac{\sqrt{\lambda t}}{\sqrt{1+\frac{R_{d}}{\lambda }}}=\frac{\lambda \sqrt{t}}{\sqrt{\lambda +R_{d}}}$$

**Figure 7:**
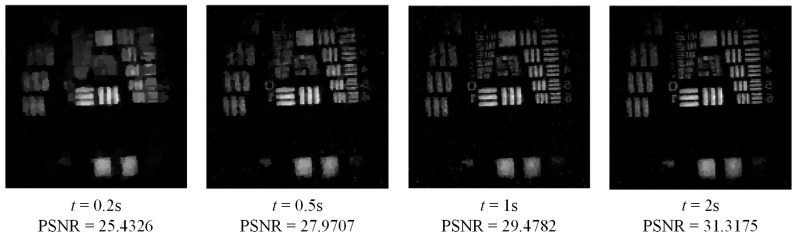
Experimental results. The size of reconstructed images is 128 × 128, and the number of measurements is 8000. The time of each measurement *t* is 0.2 s, 0.5 s, 1 s, and 2 s respectively.

From Equation (9), it can be seen that, the part that contributes the most to the SNR is $\sqrt{\lambda t}$. That is to say, the SNR mainly depends on the number of detected photons. In addition, SNR can be improved as the measurement time is made longer. In order to verify this conclusion, we set a group of experiments with different measurement time under the condition of photon counts rate 2500 cps, in which the sparse binary random matrix is selected as measurement matrix. The experimental results are shown in Figure 7. Also the PSNRs of reconstructed images are calculated for quantitative comparison of reconstructed images. The size of reconstructed images is 128 × 128, and the number of measurements is 8000. The time of each measurement is 0.2 s, 0.5 s, 1 s, and 2 s respectively.

As can be seen from Figure 7, the quality of reconstructed images is improved with the extension of measurement time *t*, which can also be confirmed by the PSNRs. So the experimental results perfectly verified the above conclusion given by Equation (9).

## 5. Conclusions

The large area imaging in extreme darkness with high sensitivity is realized. It covers the entire DMD working region with a large area photomultiplier tube single photon detector used as the bucket detector. A DMD control and photon counting logic based on FPGA is specially developed and several mirrors are combined together as one pixel to reduce sampling and reconstruction time of large-area imaging, so this imaging scheme enables important application in the large-aperture and wide-field single photon imaging systems. The properties of the constructed matrices are analyzed in theory, including sparse binary random matrix, m sequence matrix and true random number matrix. It shows that, the mutual coherence coefficient of sensing matrix decreases with the compressive sampling ratio increasing. Moreover, the coherence coefficient corresponding to m sequence matrix turns out to be smallest under the discrete wavelet transform sparse basis condition. The performance parameters of imaging system measurement matrix sparsity ratio, compressive sampling ratio and reconstruction time are experimentally verified. The experimental results also show that increasing the measurement matrix sparsity ratio and compressive sampling ratio can improve the quality of image reconstruction. When the sparsity ratio and compressive sampling ratio increase to a certain value, the image reconstruction quality tends to be saturated. In order to obtain better quality of image reconstruction at a limited acquisition and reconstruction time, the sparsity ratio is preferably 0.1, and the compression sampling ratio is preferably 0.6. The experiments of different measurement matrices show that the image reconstruction performance of m sequence matrix is better.

## Figures and Tables

**Table 1:** Evaluation indices of reconstruction image under different measurement matrices.

Evaluation Index	MSE	PSNR	MSSIM
Sparse binary random matrix	29.6249	33.3144	0.8607
m sequence matrix	16.3040	36.0078	0.9081
True random number matrix	27.3209	33.7658	0.8685
